# Effectiveness of screening and ultra-brief intervention for hazardous drinking in primary care: pragmatic cluster randomised controlled trial

**DOI:** 10.1136/bmj-2024-083985

**Published:** 2025-08-12

**Authors:** Ryuhei So, Kazuya Kariyama, Shunsuke Oyamada, Sachio Matsushita, Hiroki Nishimura, Yukio Tezuka, Takashi Sunami, Toshi A Furukawa, Ethan Sahker, Mitsuhiko Kawaguchi, Haruhiko Kobashi, Sohji Nishina, Yuki Otsuka, Hideyuki Kanda, Yasushi Tsujimoto, Yoshinori Horie, Hitoshi Yoshiji, Takefumi Yuzuriha, Kazuhiro Nouso

**Affiliations:** 1 CureApp, Tokyo, Japan; 2 Okayama Psychiatric Medical Centre, Okayama, Japan; 3 Department of Health Promotion of Human Behaviour, Kyoto University Graduate School of Medicine/School of Public Health, Kyoto, Japan; 4 Scientific Research WorkS Peer Support Group (SRWS-PSG), Osaka, Japan; 5 Department of Gastroenterology, Okayama City Hospital, Okayama, Japan; 6 Department of Biostatistics, JORTC Data Centre, Tokyo, Japan; 7 National Hospital Organization, Kurihama Medical and Addiction Centre, Yokosuka, Kanagawa, Japan; 8 Addiction Treatment Centre, Department of Psychiatry, Okinawa Rehabilitation Centre Hospital, Okinawa, Japan; 9 Saga Prefecture Medical Centre Koseikan, Saga, Japan; 10 Population Health and Policy Research Unit, Medical Education Centre, Kyoto University Graduate School of Medicine, Kyoto, Japan; 11 Kawaguchi Medical Clinic, Okayama, Japan; 12 Department of Hepatology, Japanese Red Cross Okayama Hospital, Okayama, Japan; 13 Department of Gastroenterology and Hepatology, Kawasaki Medical School, Okayama, Japan; 14 Department of General Medicine, Okayama University Graduate School of Medicine, Dentistry and Pharmaceutical Sciences, Okayama University, Okayama, Japan; 15 Department of Public Health, Graduate School of Medicine, Dentistry and Pharmaceutical Sciences, Okayama University, Okayama, Japan; 16 Oku Medical Clinic, Osaka, Japan; 17 Keiai Clinic, Tokyo, Japan; 18 Department of Gastroenterology, Nara Medical University, Nara, Japan; 19 National Hospital Organization, Hizen Psychiatric Medical Centre, Saga, Japan; 20 Chikugo Yoshii Cocoro Hospital, Fukuoka, Japan

## Abstract

**Objective:**

To evaluate the effectiveness of a doctor delivered screening and ultra-brief intervention (<1 minute) compared with simplified assessment only for reducing alcohol intake among patients with hazardous drinking in primary care.

**Design:**

Pragmatic cluster randomised controlled trial.

**Setting:**

40 primary care clinics in Japan that did not provide routine screening and brief intervention for hazardous drinking or treatment or self-help groups for alcohol dependency.

**Participants:**

1133 outpatients aged 20-74 years with hazardous drinking (AUDIT-C (alcohol use disorders identification test-consumption) scores ≥5 for men and ≥4 for women). Clinic clusters were allocated to a study arm using a computer generated random sequence. Participants and staff who collected participant reported outcomes remained blinded to assignment.

**Interventions:**

Primary care clinics were randomised to ultra-brief intervention (21 clinics, 531 patients) or simplified assessment only (19 clinics, 602 patients) groups. The intervention group comprised screening with AUDIT-C followed by brief oral advice and an alcohol information leaflet delivered in <1 minute. The control group comprised simplified assessment with AUDIT-C only.

**Main outcome measures:**

The primary outcome was total alcohol consumption in the four weeks preceding the 24 week follow-up. Secondary outcomes included total alcohol consumption in the four weeks preceding the 12 week follow-up, and readiness to change drinking behaviour, measured at 12 and 24 weeks.

**Results:**

At 24 weeks, the difference in total alcohol consumption between the ultra-brief intervention group (1046.9 g/4 weeks (g/4wk), 95% confidence interval (CI) 918.3 to 1175.4) and control group (1019.0 g/4wk, 893.5 to 1144.6) was 27.8 g/4wk (−149.7 to 205.4, P=0.75), with a Hedges’ g of 0.02 (95% CI −0.10 to 0.14). At 12 weeks, the difference in total alcohol consumption between the intervention group (1034.1 g/4wk, 919.6 to 1148.7) and control group (979.3 g/4wk, 866.1 to 1092.4) was 54.9 g/4wk (−104.1 to 213.9, P=0.49), with a Hedges’ g of 0.04 (−0.08 to 0.16).

**Conclusion:**

This trial found no evidence to support the effectiveness of a doctor delivered ultra-brief intervention for hazardous drinking compared with simplified assessment only in primary care in Japan.

**Trial registration:**

UMIN Clinical Trials Registry UMIN000051388.

## Introduction

Globally, harmful alcohol use is a major public health concern, contributing to three million deaths and 5.1% of the global burden of disease annually.^1^ Hazardous drinking, originally defined by a “quantity or pattern of alcohol use that places patients at risk for adverse consequences,”^2^ is seen in about 20% of patients in primary care settings^3^ ^4^ and is associated with a wide range of physical, mental, and social harms.^5^ ^6^ To address this global health issue, brief interventions consisting of screening and short counselling sessions have been widely recommended as an effective approach to reduce hazardous drinking in primary care settings.^7^ ^8^ ^9^

The Screening and Intervention Programme for Sensible drinking (SIPS) study, a large scale cluster randomised controlled trial, found that the group assigned to an ultra-brief intervention, comprising a leaflet with feedback on screening results, showed comparable reductions in hazardous drinking to the groups assigned to more time intensive brief interventions.^10^ ^11^ For policy makers and clinicians, the idea of widely implementing shorter, simpler, and cheaper interventions is highly appealing, given the low implementation rate of standard brief interventions owing to constraints on time.^7^ ^9^

Available evidence, however, remains inconclusive as to whether the ultra-brief intervention and more time intensive brief intervention are equally effective or equally ineffective compared with assessment only control.^12^ The results of randomised controlled trials comparing ultra-brief intervention with assessment only controls have been mixed across different settings. Ultra-brief intervention is shorter than standard brief intervention and may only include leaflets, booklets, or computer generated feedback letters.^13^ For inpatient settings, ultra-brief intervention shows a greater reduction in alcohol consumption compared with assessment only controls, and similar effects to 20-35 minutes of counselling.^14^ ^15^ Additionally, our small quasi-experimental study showed promising results, suggesting the potential effectiveness of ultra-brief intervention for alcohol consumption relative to assessment only control.^16^ Conversely, in community and emergency settings, randomised controlled trials do not support the effectiveness of ultra-brief intervention on alcohol consumption compared with assessment only controls.^13^ ^17^

As no randomised controlled trial has directly investigated the effectiveness of ultra-brief intervention over assessment only control in primary care settings, we designed and conducted a large scale pragmatic cluster randomised controlled trial in primary care settings in Japan. We evaluated the effectiveness of a doctor delivered screening and ultra-brief intervention (<1 minute) compared with a simplified assessment only control at individual participant level in reducing alcohol consumption at follow-up of 12 and 24 weeks.

## Methods

The EASY (Education on Alcohol after Screening to Yield moderated drinking) study was a pragmatic, cluster randomised, parallel group, superiority trial. In this paper we report the results in accordance with the CONSORT (Consolidated Standards of Reporting Trials) 2010 extension for cluster randomised controlled trials.^18^ Before screening began, we prospectively registered the study with the UMIN Clinical Trials Registry (https://www.umin.ac.jp/ctr/) on 23 June 2023. The prevalence of hazardous drinking among all participants who responded to the screening survey is reported elsewhere.^4^

### Settings and procedures

**Fig 1:**
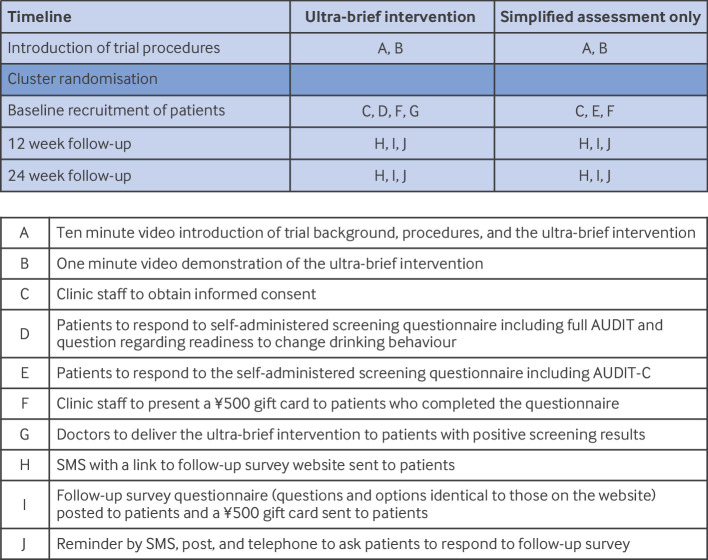
Graphical depiction of procedures through the trial. ¥500 (£2.54; €2.97; $3.42). AUDIT=alcohol use disorders identification test; AUDIT-C=alcohol use disorders identification test-consumption

Figure 1 presents a summary of the trial procedures, showing the sequence and contents of screening, intervention, and follow-up. We invited primary care clinics from urban, suburban, and rural areas across four prefectures in western Japan (Okayama, Hyogo, Osaka, and Hiroshima) through personal connections and established referrals within the local healthcare system. Based on the Japanese National Health and Nutrition Survey,^19^ these prefectures represent varying levels of alcohol consumption: Okayama is slightly below the national average, Hyogo and Hiroshima are at the national average, and Osaka is slightly above the national average. When inviting clinics, we provided videos to introduce the study procedure and show the ultra-brief intervention.

We allocated clinic clusters to the two arms of the study using a block randomisation method without any stratification or matching. The statistician (SO), who was not involved in the recruitment of clusters, generated a random sequence on a computer. Using this random sequence, another researcher (SM), also not involved in the recruitment of clusters, allocated the clusters to the study groups before each site initiated the screening and intervention.

The screening and intervention for participants took place at each cluster between 29 June and 7 August 2023. We asked each cluster to invite all eligible patients consecutively, until each cluster exceeded the target enrolment of 29 patients. Reception staff or nurses (not doctors) screened patients for basic eligibility criteria (age, pregnancy status, and covid-19-like symptoms) and invited eligible patients at the clinic reception to provide written consent for trial participation after reading the informed consent document. To keep participants blinded, the document mentioned possible brief advice from doctors but did not disclose the random allocation or intervention of interest. To replicate real world clinical settings as closely as possible, no formal oral explanation of the trial was given. The consenting participants responded to the screening survey questionnaire. Those who met the criterion for intervention and follow-up in the ultra-brief intervention group received the intervention right after screening, whereas those in the simplified assessment only group did not. Receptionists and doctors remained unblinded throughout this procedure.

We conducted follow-up surveys at 12 and 24 weeks after the screening and intervention to assess outcomes. Participants were given the option to respond to the follow-up survey questionnaires either on paper or online. The follow-up survey questionnaires and QR codes for accessing the online survey were sent by post. Additionally, text links to the online survey were sent through mobile phone short messages to maximise response rates. This dual approach ensured that participants could choose the most convenient method for completing the follow-up surveys.

For the 12 week survey, we initially used registered post that required signature on delivery; however, as this resulted in some delivery failures due to recipient absence, we switched to standard post for the 24 week survey. The mobile phone messages also included a notification about the mailed questionnaires.

The survey company, Neo marketing, contracted by the research team administered the follow-up surveys and entered the screening and follow-up survey data. If data were missing, staff of the survey company tried to contact participants through text messages or phone calls to obtain data. Staff members responsible for missing data collection remained blinded to the allocation of trial participants.

### Eligibility criteria for participants and clusters

Inclusion criteria for clusters were primary care clinics in Japan that had not previously screened patients with the alcohol use disorders identification test-consumption (AUDIT-C)^20^ and brief intervention for hazardous drinking in routine practices, not used specialised treatment for alcohol dependence, or not provided opportunities for self-help groups focused on alcohol dependence.

Patients were considered eligible for screening if they were outpatients aged 20 to 74 years and able to understand the informed consent form and provide written consent. Exclusion criteria at screening were pregnancy, suspected covid-19, and unsuitability for the study according to the doctors at the clusters. An inclusion criterion for follow-up was hazardous drinking, defined as AUDIT-C scores ≥5 for men and ≥4 for women at the screening survey.

### Measures

#### Screening survey

For all screened participants at both intervention and control clinics, we obtained data on age group, sex, medical history (collected using a checklist of common medical conditions related to alcohol consumption in the questionnaire), type of visit (eg, first, as needed, or routine appointment), and readiness to change diet and smoking behaviour.

We included assessments of diet and smoking behaviour to mask the study hypotheses related to drinking behaviour. To assess participants’ readiness to change diet and smoking behaviour, we used questions from the Japanese National Health and Nutrition Survey.^19^ The response options were combined and truncated to reduce the burden on respondents. To identify participants engaging in hazardous drinking, we used AUDIT-C (scores 0-12, with higher scores representing more hazardous drinking), which comprises questions 1 to 3 of the alcohol use disorders identification test (AUDIT)^20^ and has been validated for screening use in primary care settings.^21^ A validation study in Japan found that a score on AUDIT-C of ≥4 for women and ≥5 for men corresponds to a score of ≥8 on AUDIT, indicating hazardous drinking or potential alcohol dependence.^20^ ^22^ We calculated the baseline total alcohol consumption in grams per four weeks based on the first and second items of the AUDIT-C questionnaire.

Additionally, the questionnaire given to screening participants at the ultra-brief intervention clusters included questions about readiness to change drinking behaviour, along with questions 4 to 10 of AUDIT. We evaluated the readiness to change drinking behaviour with the question, “Are you considering improving your drinking habits?,” with the following response options: “Already working on improvement,” “Intending to improve,” “Interested but no intention to improve,” “No intention to improve,” and “No improvement needed.” We developed these question and response options based on the Japanese National Health and Nutrition Survey,^19^ although their psychometric reliability and validity have not yet been confirmed. We did not include the assessment of readiness to change drinking behaviour in the simplified assessment only cluster questionnaires, as the assessment itself might influence behavioural change, potentially making it more difficult to detect the effects of ultra-brief intervention.^23^ ^24^

After the screening survey period, we gathered information from the clusters on the approximate percentages of all potential participants who were asked if they might participate in the EASY study and agreed. The details on these percentages are reported elsewhere.^4^

#### Follow-up survey

We conducted follow-up surveys at 12 and 24 weeks after the screening survey to obtain data on amount of alcohol consumption and readiness to change diet and smoking and drinking behaviours from all trial participants. To assess the amount of alcohol consumption, we developed a set of questions asking participants about their usual drinking behaviour, including the type of alcoholic beverages consumed, type of containers (eg, cans or glasses), number of glasses, bottles, or cans of alcoholic beverage consumed (“When drinking, how much do you typically consume?”), and frequency of drinking days (“How often do you drink alcohol?”), with response options ranging from “Never” and “Less than once a month” to “Six times a week” and “Daily.”

In terms of readiness to change health related lifestyles, the questionnaires were the same as those used at the screening survey. However, for the question on readiness to change drinking behaviour, the option, “Interested but no intention to improve” was inadvertently omitted from the follow-up survey.

#### Outcomes

The primary outcome was total alcohol consumption in the four weeks preceding the 24 week follow-up (total alcohol consumption is equivalent to the average alcohol consumption specified as the primary outcome in the trial registration). Using the detailed responses to the follow-up survey questionnaires, we calculated the total alcohol consumption by referring to a table listing the average alcohol content by volume for various types of beverages, and the standard volumes of different containers. The total alcohol consumption (g/4 weeks (g/4wk)) was calculated using the formula:

Volume (mL)×alcohol by volume/100×number of glasses, bottles, or cans×0.8×frequency of drinking days

The initial version of this table was created before the follow-up survey. If participants’ responses included beverages or containers not listed in the table, we defined the alcohol by volume and standard volume without considering the participant’s allocated group.

The method used to measure amount of alcohol consumption as our primary outcome was chosen after careful consideration of the balance between capacity for behavioural modification and sensitivity to detect minor effects, as well as validity of the metric. While more detailed measures such as drinking diaries or timeline followback^25^ could provide information about frequency and binge drinking patterns, these methods would require considerable effort from participants, or extended interaction with researchers. These approaches therefore could constitute more intensive interventions than the intended ultra-brief intervention for one minute. Conversely, calculating alcohol consumption based on AUDIT items 1 and 2, which assess drinking frequency and quantity using 5 point scales, would be too crude to detect the small effect size expected from ultra-brief intervention. In addition, although AUDIT-C shows a reasonably high correlation with timeline followback (ρ=0.75),^26^ item 1 limits responses to ≥4 days each week and item 2 limits responses to ≥10 drinks, suggesting a ceiling effect.^27^

Additionally, the World Health Organization (WHO) drinking risk level was calculated based on total alcohol consumption.^28^ WHO drinking risk level categorises drinking patterns into different risk levels based on total alcohol consumption daily. Low risk is defined as ≤40 g/day for men and ≤20 g/day for women; medium risk as 41-60 g/day for men and 21-40 g/day for women; high risk as 61-100 g/day for men and 41-60 g/day for women; and very high risk is >100 g/day for men and >60 g/day for women. These thresholds signify the potential health risks associated with alcohol consumption and provide guidelines for reducing such risks.

Total alcohol consumption and drinking risk level, which can be calculated from total alcohol consumption, are meaningful surrogate markers that reflect patients’ health and quality of life. Secondary analyses of randomised controlled trials for treatment of alcohol use disorder have shown that reductions in drinking risk levels achieved during treatment are maintained for up to three years,^29^ with decreases in drinking risk levels predicting fewer alcohol related consequences and improved mental health.^30^ Moreover, a large scale study of people with high risk drinking levels found that reductions in drinking risk levels were associated with a lower risk of new onset liver disease and fewer positive AUDIT-C screening results.^31^

The secondary outcomes included total alcohol consumption in the four weeks preceding the 12 week follow-up, as well as readiness to change drinking behaviour, measured at both 12 weeks and 24 weeks. For readiness to change drinking behaviour, we combined “Interested but no intention to improve” and “No intention to improve” into “No intention to improve” owing to the inadvertent omission of “Interested but no intention to improve” from the follow-up survey questionnaire. We then converted each option to numerical values: 4 for “Already working on improvement,” 3 for “Intending to improve,” 2 for “No intention to improve,” and 1 for “No improvement needed.”

To determine the specificity of the intervention effects on readiness to change drinking behaviour, we also included readiness to change diet and smoking behaviour, measured at 12 and 24 weeks, as secondary outcomes. For readiness to change diet, we converted each option to numerical values: 4 for “Already working on improvement,” 3 for “Intending to improve,” 2 for “No intention to improve,” and 1 for “No improvement needed.” For readiness to change smoking behaviour, among participants who were current smokers at baseline, we converted the options to numerical values: 3 for “Smoked but quit,” 2 for “Intending to improve,” and 1 for “No intention to improve.”

#### Doctor adherence to ultra-brief intervention

At cluster level, we asked doctors in the ultra-brief intervention clusters to mark the screening survey questionnaire for each patient to confirm they had provided an oral feedback message on the screening survey and an alcohol information leaflet as our measure of doctor adherence to the ultra-brief intervention procedure. Additionally, in the follow-up survey questionnaire we included a question asking participants, “Did you receive advice about your drinking habits from the physician during the consultation on the day you participated in the survey in July-August 2023?” We categorised the responses as: “Received,” participant answered, “Received” at either the 12 week or the 24 week follow-up; “Did not receive,” participant answered, “Did not receive” at either the 12 week or the 24 week follow-up or at any point; and “Unsure,” participant did not answer “Received” or “Did not receive” at any time.

### Intervention and control

#### Ultra-brief intervention group

In the intervention clinics, all eligible patients were screened with AUDIT. Those who met the AUDIT-C inclusion criterion received the ultra-brief intervention. At the clinic reception, screening participants responded to AUDIT and the questionnaire that included readiness to change their drinking behaviour. After the screening, trial participants received an alcohol information leaflet and brief oral message from their doctor at the end of the consultation. The contents of the ultra-brief intervention were developed based on the materials and procedures used in our pilot trial in a general hospital setting, which demonstrated promising results.^16^ We designed both the leaflet and the brief oral message in accordance with the FRAMES (feedback, responsibility, advice, menu of options, empathy, and self-efficacy) approach.^32^

The double sided leaflet is in a simple and compact format, using easy-to-understand illustrations and colours (see supplementary files 1-4). It provides advice on moderated drinking levels and tips for reducing alcohol consumption. The contents comprised an explanation of the “standard drink” unit used to measure alcohol consumption and a conversion table for various types of alcoholic beverages; guidelines for drinking levels, health risks associated with excessive drinking, and the health benefits of reducing alcohol intake; specific steps for reducing alcohol consumption, including setting goals, recording progress, and self-evaluation; and tips for avoiding excessive drinking and an introduction to tools, including a website and a smartphone app.

The template of the oral message was: “Mr./Ms. [Name], you might drink too much.” (Feedback); “I recommend you calculate your alcohol consumption on your own.” (Advice); “The information here (in the leaflet) will be beneficial for you.” (chance of effectiveness); “You can easily apply these recommendations (in the leaflet) starting today.” (Assurance of feasibility); and “I look forward to hearing your thoughts when we meet next time” (Motivation through commitment).

The health belief model^33^ framework may offer a theoretical rationale for effectiveness of ultra-brief intervention, suggested by empirical evidence.^10^ ^14^ ^15^ ^16^ Physical symptoms and test results from internal medicine visits heighten perceived susceptibility to alcohol related problems, creating a teachable moment. The leaflet reinforced perceived severity by providing information on health impacts and emphasised the benefits of appropriate drinking while reducing perceived barriers through specific guidelines.

Doctors in the ultra-brief intervention clusters were given a paper outlining the procedure for conducting the ultra-brief intervention, along with a demonstration video.

#### Simplified assessment only group

In the control clinics, to better align the control condition with routine clinical practice, which typically does not involve systematic screening for drinking behaviour, we asked all eligible participants to fill in a simplified questionnaire. This simplified questionnaire included the same items used in the ultra-brief intervention group but excluded AUDIT questions 4 to 10 and the item to assess readiness to change drinking behaviour. We removed these items because previous studies have suggested that completing AUDIT or similar assessments of drinking behaviour can lead to improvements in drinking behaviour.^23^ ^24^

After completing the screening questionnaire, participants submitted their questionnaire responses to the reception, and the receptionist stored the questionnaires in a box to be sent back to the research centre. Therefore, the doctors in the simplified assessment only clusters were unaware of the questionnaire responses and provided usual care to the participants.

### Sample size calculation

We set the sample size for the cluster randomised controlled trial at 1125 participants. This was determined based on an assumed effect size of 0.25 standardised mean difference for the ultra-brief intervention, with a two sided α level of 0.05, a power of 0.80, 40 clusters, and an intracluster correlation coefficient between 0.03 and 0.04.^10^ Under these assumptions, we calculated the sample size necessary to detect the effect of ultra-brief intervention to be between 800 and 1000 participants. Taking the median of this range, we estimated 900 participants for the cluster randomised controlled trial. Assuming a dropout rate of 20% during the follow-up period of the randomised controlled trial, the necessary sample size increased to 1125 participants. To achieve the sample size, we instructed each cluster to conduct the screening survey until a minimum of 29 participants with AUDIT-C scores of ≥5 for men and ≥4 for women was reached.

### Statistical analysis

We used SAS software version 9.4 (SAS Institute, Cary, NC) and R version 4.2.3.^34^ The analysis included two populations: the intention-to-treat (ITT) population and the per protocol population. The ITT population consisted of all participants, who were analysed in the group to which they had been randomised. The per protocol population included participants who received the ultra-brief intervention based on the doctor’s report in the ultra-brief intervention group and all participants in the simplified assessment only group.

The primary analysis was conducted on the ITT population, but the same analysis was also performed on the per protocol population. A general linear mixed effects model was used to estimate the least square means of total alcohol consumption in the past four weeks for each group at each time point, along with the point estimates and 95% confidence intervals (CIs) for the differences between groups.^35^ The dependent variable was the total alcohol consumption. The fixed effects in the model included study group (ultra-brief intervention versus simplified assessment only), time point (12 weeks, 24 weeks), baseline total alcohol consumption converted from AUDIT-C score, the group×time point interaction, and participant characteristics at screening (sex, quantified age group (converted to a continuous variable by assigning median values to each group)), and AUDIT-C score. The model considered the cluster as a random effect (random intercept). An unstructured variance-covariance matrix was assumed for the correlation between time points for the dependent variable, and the Kenward-Roger method was used to calculate the degrees of freedom. We analysed observed cases only, without imputation for missing data, under the assumption that missing data occurred at random.^36^ ^37^ We also calculated Hedges’ g based on the between group differences and pooled standard deviations at each follow-up time point.

We conducted a post hoc sensitivity analysis using instrumental variable methods to adjust for non-adherence of doctors in intervention delivery (a common issue in pragmatic trials) and estimate the local average treatment effect among the subpopulation of those who adhered to intervention delivery.^38^ ^39^ ^40^ We used an adjusted cluster level approach with two stage least squares estimation,^41^ where randomisation assignment served as the instrumental variable and participant reported recognition of receiving advice as the endogenous variable. We excluded participants with missing data on the outcomes and the endogenous variable. In the simplified assessment only group, we classified all analysed participants as not having received advice because we did not provide leaflets to clinics, and doctors did not review screening survey results. Cluster level summaries were adjusted for individual level baseline covariates before performing two stage least squares regression. We applied small sample degrees of freedom correction for inference.

In open cluster randomisation, selective screening of patients could affect the distribution of potential effect modifiers between groups. Therefore, we performed another post hoc sensitivity analysis that replicated the primary analysis while excluding participants from clusters that selectively invited patients to the screening survey.

For the analysis of readiness to change drinking behaviour, we used a general linear mixed effects model similar to total alcohol consumption in the ITT population. The dependent variable was the quantified readiness to change drinking behaviour, and the independent variables included the group, time point, group×time point interaction, sex, quantified age group, and baseline total alcohol consumption converted from AUDIT-C score. Baseline readiness to change drinking behaviour was not included as a covariate because it was not collected in the simplified assessment only group. Similar models were used for analyses of diet and smoking behaviour, with their respective baseline readiness to change scores as covariates instead of total alcohol consumption.

To examine potential effect modifiers, we performed exploratory subgroup analyses for both total alcohol consumption and readiness to change drinking behaviour in the ITT population. The subgroup variables included AUDIT-C score category (4-6, 7-9, 10-12), representing equal thirds of the possible score range, sex (men, women), age group (20-39, 40-49, 50-59, 60-69, 70-79 years), and visit type (routine appointment or otherwise) collected at screening survey. We illustrated the results of the subgroup analyses using forest plots.

We aggregated doctor reported adherence with the ultra-brief intervention at screening, WHO drinking risk level, readiness to change drinking behaviour, and participant reported recognition of receiving advice at follow-up by allocation group.

### Ethical considerations

To closely mimic real world clinical settings, potential participants were asked to provide written consent for trial participation after reading the informed consent document, without receiving a formal oral explanation. Additionally, to minimise the risk of expectation bias, we only mentioned that the study was related to health related lifestyles, as explicitly mentioning alcohol use could affect participants’ responses and behaviours. This decision balanced transparency with methodological rigor. We did not ask clinics to restrict their usual clinical discussions about alcohol related problems with patients.

### Changes from the protocol and the statistical analysis plan

The statistical analysis plan version 1.0 was finalised on 20 March 2024, before conducting any statistical analyses. This version included descriptive statistics for baseline characteristics, estimation of between group differences in total alcohol consumption, and readiness to change drinking behaviour using general linear mixed effects models, and their corresponding subgroup analyses. On 28 November 2024, we revised the statistical analysis plan to align with the Guidelines for the Content of Statistical Analysis Plans in Clinical Trials.^42^ During the peer review process, we added sensitivity analyses accounting for non-adherence of doctors and selective screening procedure.

Of note, among the outcomes analysed, only total alcohol consumption was prespecified in the protocol, statistical analysis plan version 1.0, and trial registry. Analyses of readiness to change drinking behaviour, as well as diet and smoking behaviour, were inadvertently omitted from both the protocol and the trial registration and are therefore considered post hoc analyses. Categorical summaries of total alcohol consumption and readiness to change drinking behaviour were performed as post hoc analyses not specified in the statistical analysis plan version 1.0.

### Patient and public involvement

Although this trial did not formally incorporate patient and public involvement (PPI) in its protocol, we conducted informal consultations with two male outpatients (in their 60s and 70s) on 11 May 2023, during the study design phase, which led to improvements in questionnaire readability. The limited implementation of PPI was due to time constraints within the grant period and lack of formal PPI training among our research team. Following peer review, we sought feedback on our manuscript from a representative of Alcohol yakubutsu mondai zenkoku Shimin Kyoukai (ASK; a Japan-wide non-governmental organisation on alcohol and substance issues),^43^ a non-profit corporation, to prevent problems related to addiction to alcohol, other substances, or behaviour, who has lived experience as a family member of someone with alcohol dependence.

## Results

**Fig 2:**
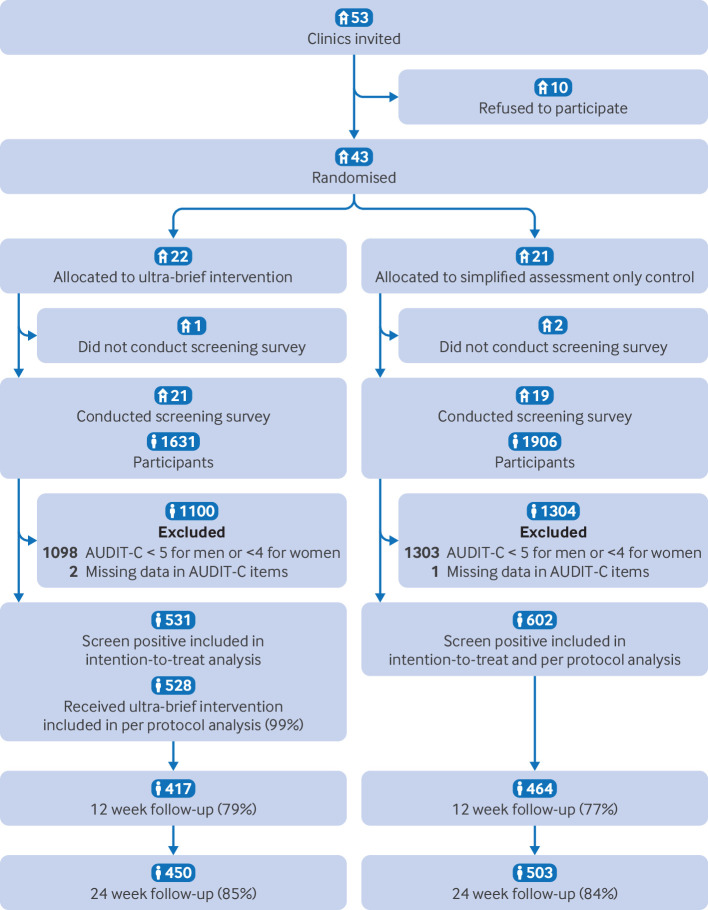
Flow chart of clusters and patients through the trial. The 12 week survey was sent by registered post and required signature, resulting in some delivery failures when recipients were absent. The 24 week survey was therefore sent by standard post. AUDIT-C=alcohol use disorders identification test-consumption

Figure 2 shows the flow of participants through the trial. We did not exclude any clinics invited to take part, regardless of whether they provided screening, brief interventions, or more intensive interventions in their routine practices. Recruitment of participants occurred from 29 June to 7 August 2023 across 43 clinics, although three clinics did not initiate the study and therefore did not recruit or screen any participants.

Of the 40 clinics that conducted the screening survey (ultra-brief intervention: 21 clinics, simplified assessment only: 19 clinics), four clinics (three and one, respectively) only invited outpatients suspected of having drinking problems to participate in the screening survey, whereas the remaining 36 clinics attempted to invite outpatients consecutively. Most clinics invited nearly all eligible patients (ultra-brief intervention: median 90% (interquartile range (IQR) 55-100%), simplified assessment only: 95% (70-100%)), although some clinics were unable to maintain consecutive recruitment during periods of high workload. Finally, because some patients declined participation, the median percentage of eligible patients included in screening at each clinic was around 70% (ultra-brief intervention: median 76% (IQR 52-90%), simplified assessment only: 71% (65-98%)). Detailed results of the screening survey are reported elsewhere.^4^

In total, 3537 participants from 40 clinics consented to participate: 1631 in the ultra-brief intervention group and 1906 in the simplified assessment only group. After excluding those who did not meet the hazardous drinking criteria of AUDIT-C or with missing data in AUDIT-C items, 531 participants in the ultra-brief intervention group and 602 in the simplified assessment only group were eligible for follow-up. Among the ultra-brief intervention group, 528 (99%) participants who received the intervention adhered to the protocol. Overall, 473 (89%) participants in the ultra-brief intervention group and 535 (89%) in the control group provided complete data on total alcohol consumption, with 417 (79%) and 464 (77%), respectively, providing data at 12 weeks and 450 (85%) and 503 (84%) providing data at 24 weeks.

Table 1 presents the baseline characteristics of the participants. The ultra-brief intervention group had a higher proportion of women than the control group (36% *v* 31%). Additionally, the mean total alcohol consumption, calculated using AUDIT-C, was slightly higher in the ultra-brief intervention group compared with control group (749.9 g/4wk *v* 721.9 g/4wk).

**Table 1:** Baseline characteristics of participants with hazardous drinking randomly allocated to receive ultra-brief intervention (<1 minute) or simplified assessment only (control). Values are number (percentage) unless stated otherwise

Characteristics	Ultra-brief intervention (n=531)	Simplified assessment only (n=602)	Total (n=1133)
Age group (years):			
20-29	17 (3)	27 (4)	44 (4)
30-39	40 (8)	33 (5)	73 (6)
40-49	89 (17)	79 (13)	168 (15)
50-59	118 (22)	150 (25)	268 (24)
60-69	170 (32)	190 (32)	360 (32)
70-74	97 (18)	123 (20)	220 (19)
Mean (SD) age* (years)	57.3 (12.6)	58.0 (12.7)	57.6 (12.6)
% men	342 (64)	417 (69)	759 (67)
Visit type:			
First	26/527 (5)	41/596 (7)	67/1123 (6)
Routine appointment	396/527 (75)	456/596 (77)	852/1123 (76)
As needed	105/527 (20)	99/596 (17)	204/1123 (18)
Smoker	165/530 (31)	168/601 (28)	333/1131 (29)
Medical history:			
Hypertension	287 (54)	347 (58)	634 (56)
Hyperuricaemia	74 (14)	62 (10)	136 (12)
Diabetes	75 (14)	88 (15)	163 (14)
Dyslipidaemia	97 (18)	114 (19)	211 (19)
Liver diseases	60 (11)	63 (10)	123 (11)
Digestive diseases	88 (17)	105 (17)	193 (17)
Mean (SD) AUDIT-C score	7.3 (2.4)	7.2 (2.3)	7.2 (2.3)
Mean (SD) baseline TAC based on AUDIT-C score (g/4wk)	749.9 (525.5)	721.9 (516.4)	735.0 (520.1)

Unless otherwise specified, the denominator for percentages is the total sample size shown at the top of each column. When the denominator differs, it is indicated in that row.

AUDIT-C=alcohol use disorders identification test-consumption; SD=standard deviation, TAC=total alcohol consumption.

* Categorical variable age group was converted into a continuous variable using the median value of each range and then calculating the mean.

### Total alcohol consumption at follow-up

**Fig 3:**
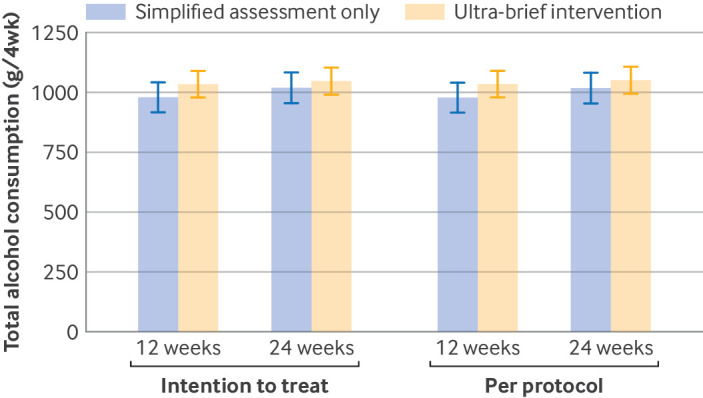
Total alcohol consumption (grams in four weeks (g/4wk) before follow-up) at 12 and 24 weeks. Whiskers represent standard errors

Table 2 and figure 3 present the total alcohol consumption at 12 and 24 weeks for the ITT and per protocol populations, showing least square means, group differences, and Hedges’ g estimated from general linear mixed effects models. In the ITT population, the primary outcome of total alcohol consumption at 24 weeks was 1046.9 g/4wk (95% CI 918.3 to 1175.4) in the ultra-brief intervention group and 1019.0 g/4wk (893.5 to 1144.6) in the control group. The intracluster correlation coefficient for total alcohol consumption of 0.04 was as expected from the sample size calculations, which assumed a range of 0.03-0.04. The difference between groups was 27.8 g/4wk (95% CI −149.7 to 205.4), with a Hedges’ g of 0.02 (95% CI −0.10 to 0.14).

**Table 2:** Total alcohol consumption at 12 week and 24 week (primary outcome) follow-up in participants with hazardous drinking randomly allocated to receive ultra-brief intervention (<1 minute) or simplified assessment only (control)

Follow-up by dataset
Total alcohol consumption (g/4wk)
Difference (95% CI)
P value
Hedges’ g* (95% CI)
Ultra-brief intervention
Simplified assessment only
Intention to treat:
n=531
n=602


12 weeks
1034.1 (919.6 to 1148.7)
979.3 (866.1 to 1092.4)
54.9 (−104.1 to 213.9)
0.49
0.04 (−0.08 to 0.16)
24 weeks
1046.9 (918.3 to 1175.4)
1019.0 (893.5 to 1144.6)
27.8 (−149.7 to 205.4)
0.75
0.02 (−0.10 to 0.14)
Per protocol:
n=528
n=602


12 weeks
1034.4 (918.5 to 1150.3)
978.0 (863.6 to 1092.4)
56.4 (−104.4 to 217.2)
0.48
0.04 (−0.08 to 0.16)
24 weeks
1050.6 (920.9 to 1180.4)
1017.8 (891.2 to 1144.5)
32.8 (−146.5 to 212.1)
0.72
0.02 (−0.10 to 0.14)
**Sensitivity analyses**
Doctor non-adherence to intervention delivery:
n=450
n=503


12 weeks
1024.2 (831.01 to 1217.28)
994.8 (914.9 to 1074.62)
29.4 (−170.1 to 228.9)
0.77
0.03 (−0.16 to 0.22)
24 weeks
1116.7 (786.0 to 1447.4)
1018.0 (922.5 to 1113.5)
98.7 (−242.9 to 440.3)
0.56
0.08 (−0.19 to 0.35)
Selective screening:
n=434
n=571


12 weeks
1059.7 (931.5 to 1187.8)
986.3 (868.2 to 1104.5)
73.4 (−98.8 to 245.5)
0.39
0.05 (−0.07 to 0.18)
24 weeks
1087.7 (943.6 to 1231.8)
1026.5 (895.0 to 1158.0)
61.1 (−131.8 to 254.1)
0.53
0.04 (−0.08 to 0.16)

A linear mixed model estimated least square means with study group, time point, baseline total alcohol consumption (converted from AUDIT-C score), group×time point interaction, and participant characteristics (sex, age group, and AUDIT-C score) as fixed effects, considering the cluster as a random effect.

AUDIT-C=alcohol use disorders identification test-consumption; CI=confidence interval.

* Adjusted standardised mean difference effect size of alcohol intake (grams in four weeks (g/4wk) before follow-up).

Table 2 also includes results from the sensitivity analysis accounting for non-adherence of doctors to intervention delivery, which showed a difference between groups of 98.7 g/4wk (95% CI −242.9 to 440.3) at 24 weeks. Additionally, the sensitivity analysis accounting for selective screening showed a difference between groups of 61.1 g/4wk (−131.8 to 254.1) at 24 weeks.

When categorising total alcohol consumption by WHO drinking risk levels in the ITT population at 24 weeks, 263 participants (58%) in the ultra-brief intervention group were classified as abstinent or low risk and 108 (24%) as medium risk. In the control group, 307 (61%) participants were classified as abstinent or low risk and 109 (22%) as medium risk (table 3).

**Table 3:** World Health Organization drinking risk level at follow-up in intention-to-treat dataset of participants with hazardous drinking randomly allocated to receive ultra-brief intervention (<1 minute) or simplified assessment only (control). Values are number (percentage) unless stated otherwise

Drinking risk level	Ultra-brief intervention	Simplified assessment only	P value
12 weeks:	n=417	n=464	
Abstinent or low risk	245 (59)	264 (57)	0.9
Medium risk	101 (24)	122 (26)	
High risk	48 (12)	50 (11)	
Very high risk	23 (5.5)	28 (6.0)	
24 weeks:	n=450	n=503	
Abstinent or low risk	263 (58)	307 (61)	0.8
Medium risk	108 (24)	109 (22)	
High risk	53 (12)	56 (11)	
Very high risk	26 (5.8)	31 (6.2)	

### Readiness to change drinking behaviour, diet, and smoking behaviour at follow-up

**Fig 4:**
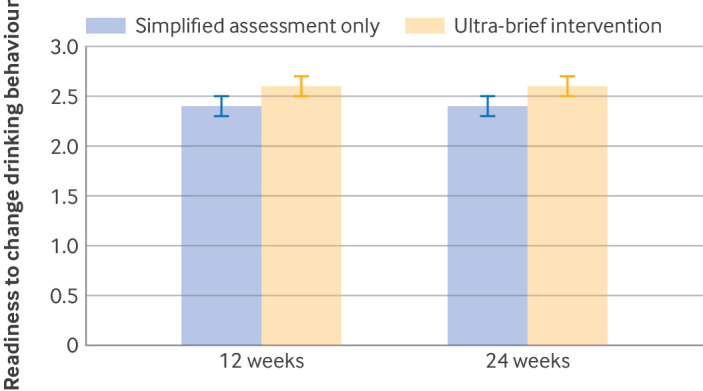
Readiness to change drinking behaviour, at 12 and 24 weeks. Whiskers represent standard errors

Readiness to change drinking behaviour, converted into numerical scores (higher scores indicating greater readiness to change), was higher in the ultra-brief intervention group compared with control group at both 12 weeks (difference 0.25 (95% CI 0.12 to 0.39); Hedges’ g 0.21 (95% CI 0.10 to 0.33)) and 24 weeks (difference 0.19 (0.05 to 0.32); Hedges’ g 0.16 (0.05 to 0.28)) (table 4, fig 4). Additionally, at 12 weeks 192 (46%) participants in the ultra-brief intervention group reported “Intending to improve” compared with 174 (37%) in the control group, and 68 (16%) versus 47 (10%), respectively, reported “Already working on improvement.” At 24 weeks, 197 (44%) participants in the ultra-brief intervention group and 206 (41%) in the control group reported “Intending to improve,” while 78 (17%) in the ultra-brief intervention group and 53 (10%) in the control group reported “Already working on improvement” (table 5).

**Table 4:** Readiness to change drinking behaviour in intention-to-treat dataset of participants with hazardous drinking randomly allocated to receive ultra-brief intervention (<1 minute) or simplified assessment only (control) at 12 week and 24 week follow-up. Values are numerical scores unless stated otherwise*

Follow-up	Ultra-brief intervention	Simplified assessment only	Difference (95% CI)	P value	Hedges’ g† (95% CI)
12 weeks	2.62 (2.52 to 2.72)	2.37 (2.27 to 2.47)	0.25 (0.12 to 0.39)	<0.01	0.21 (0.10 to 0.33)
24 weeks	2.61 (2.51 to 2.71)	2.42 (2.32 to 2.52)	0.19 (0.05 to 0.32)	<0.01	0.16 (0.05 to 0.28)

CI=confidence interval.

* Higher scores indicate greater readiness to change.

† Adjusted standardised mean difference effect size of the quantified readiness to change drinking behaviour.

**Table 5:** Proportion of participants in intention-to-treat dataset of participants with hazardous drinking randomly allocated to receive ultra-brief intervention (<1 minute) or simplified assessment only (control) with readiness to change drinking behaviour, by category. Values are number (percentage) unless stated otherwise

Follow-up by group	No	No improvement needed	No intention to improve	Intending to improve	Already working on improvement
**Ultra-brief intervention**					
Baseline	529	137 (26)	130 (24)	166 (31)	96 (18)
12 weeks	418	58 (14)	100 (24)	192 (46)	68 (16)
24 weeks	449	67 (15)	107 (24)	197 (44)	78 (17)
**Simplified assessment only**					
Baseline					
12 weeks	465	97 (21)	147 (32)	174 (37)	47 (10)
24 weeks	507	100 (20)	148 (29)	206 (41)	53 (10)

Mean scores for readiness to change diet were 2.83 in the ultra-brief intervention group and 2.73 in the control group at 12 weeks (difference 0.10 (95% CI −0.02 to 0.20)), and 2.89 and 2.86, respectively, at 24 weeks (difference 0.03 (−0.08 to 0.14)). For readiness to change smoking behaviour among smokers at baseline, mean scores were 1.75 in the ultra-brief intervention group and 1.73 in the control group at 12 weeks (difference 0.02 (−0.11 to 0.14)) and 1.79 and 1.86, respectively, at 24 weeks (difference −0.08 (−0.21 to 0.05)).

### Subgroup analysis

**Fig 5:**
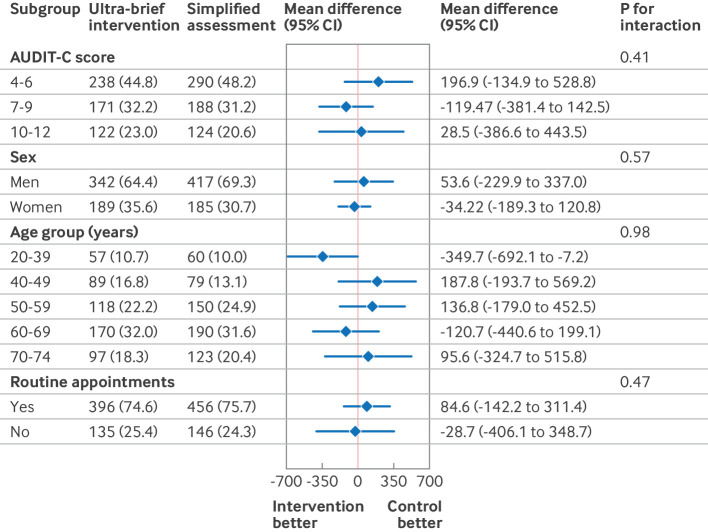
Subgroup analysis on total alcohol consumption (grams in four weeks (g/4wk) before follow-up) in participants with hazardous drinking according to study arm, at 24 week follow-up. AUDIT-C=alcohol use disorders identification test-consumption; CI=confidence interval

**Fig 6:**
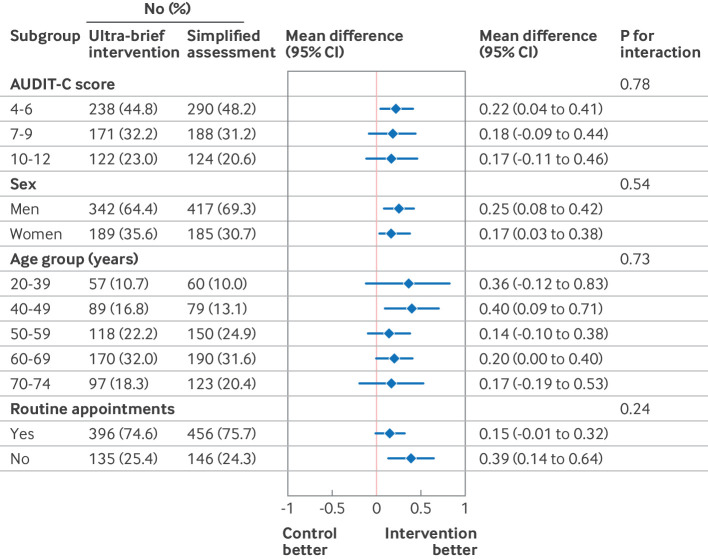
Subgroup analysis on readiness to change drinking behaviour, at 24 week follow-up. AUDIT-C=alcohol use disorders identification test-consumption; CI=confidence interval

Figure 5 and figure 6 present the results of the subgroup analysis in the ITT population at 24 weeks for total alcohol consumption and readiness to change drinking behaviour. No evidence was found for interactions between any of the outcomes and the subgroup variables.

### Participant reported recognition of receiving advice

Of 475 participants in the ultra-brief intervention group, 306 (64%) reported they had received advice from their doctor, whereas 147 (31%) reported that they had not. The remaining 22 participants (5%) reported they were unsure whether they had received advice.

## Discussion

The findings of this trial did not support the effectiveness of ultra-brief intervention on total alcohol consumption at 12 and 24 weeks compared with simplified assessment only among patients with hazardous drinking in Japanese primary care settings. The point estimate for difference between the groups in total alcohol consumption at 24 weeks was 27.8 g/4wk, corresponding to a Hedges’ g of 0.02, in favour of the simplified assessment only group. This finding remained consistent even in the sensitivity analyses accounting for non-adherence of doctors to intervention delivery and selective screening procedure. While total alcohol consumption values appeared higher at 12 and 24 weeks compared with baseline AUDIT-C-derived total alcohol consumption, this likely reflects the ceiling effect of AUDIT-C when measuring amount of alcohol consumption rather than actual increases in consumption.^27^ However, readiness to change drinking behaviour was more favourable in the ultra-brief intervention group at both 12 weeks and 24 weeks. The absence of similar improvements in readiness to change diet or smoking behaviour suggests the ultra-brief intervention specifically affected readiness to change drinking behaviour. These findings challenge the syllogistic reasoning that ultra-brief intervention would be effective based on the reported effectiveness of standard brief intervention^8^ and the assumed equivalence between the two approaches.^10^ Nevertheless, the ultra-brief intervention may have potential benefits in affecting patients’ readiness to change that may be particularly relevant in the context of continuous care.

### Possible reasons for null findings

Contrary to our expectation, our results did not support the effectiveness of ultra-brief intervention in primary care settings. Our pilot trial, which investigated the same ultra-brief intervention but in general hospital wards, had shown promising results.^16^ The SIPS trial in UK primary care settings did not find superiority of five minute or 20 minute brief interventions over ultra-brief intervention.^10^ These findings led us to anticipate the effectiveness of ultra-brief interventions in primary care. However, assuming our findings are correct, questions may arise about the effectiveness of both ultra-brief interventions and brief interventions compared with assessment only control in reducing alcohol consumption within a Japanese population. Alternatively, it may also mean that the Japanese population requires different motivational considerations in future iterations of brief intervention programmes. However, the possibility that our results are false negatives due to systematic or random errors should be considered.

Systematic errors could arise from factors concerning participants, training intensity, intervention adherence, and control conditions. The effectiveness of ultra-brief intervention may be diminished in participants with higher severity of drinking problems. Evidence supporting the effectiveness of brief interventions for people with alcohol dependence or heavy drinking is limited.^44^ However, our subgroup analysis did not provide evidence of effect modification for total alcohol consumption by AUDIT-C score category.

The low intensity of the training for doctors, which involved only watching a video, might have reduced the effect of the ultra-brief intervention owing to insufficient confidence or motivation of the doctors in dealing with alcohol related issues. However, lack of time among healthcare professionals is often cited as a barrier to implementation of brief interventions.^9^ Therefore, even if more intensive training could improve the effect of the intervention, it might also reduce its broad implementation, leading to a lesser impact on public health.

The possibility that the intervention was not delivered as intended has been suggested as a reason for the null findings of trials.^45^ Although the doctors reported delivering the ultra-brief intervention to most of the participants, only two thirds of those who responded to follow-up surveys in the ultra-brief intervention group perceived they had received advice from their doctors, indicating a potential issue. However, the proportion of participants who perceived they had received advice might be considered an indicator of the intensity of ultra-brief intervention rather than that of doctors’ non-adherence. Even in a pilot study conducted in a general hospital, where the researcher who developed the ultra-brief intervention administered the intervention to all participants and showed promising results, only 70% of participants in the intervention group reported that the doctor gave feedback.^16^ Given these proportions, it is likely that many participants who perceived they had not received advice actually did receive it. However, in this non-treatment seeking population, the fact that ultra-brief intervention improved readiness to change drinking behaviour represents a meaningful step towards behaviour change, even though it did not immediately lead to reduced alcohol consumption.

Too strong control conditions can lead to false negative findings in randomised controlled trials of brief interventions. Even screening itself may have some effect over no treatment at all and mask the effect of the brief interventions being tested.^23^ ^24^ However, we made the screening survey questionnaire for the control simplified assessment only group as simple as possible. If the intervention does not show superiority over this control, its effect can be considered to have not reached the level of clinical importance.

Random error may have resulted in a greater baseline total alcohol consumption in the ultra-brief intervention group, potentially leading to false negative results. The baseline total alcohol consumption, converted from AUDIT-C scores, was about 30 g/4wk higher in the ultra-brief intervention group compared with control group. Additionally, AUDIT-C has a ceiling effect when measuring total alcohol consumption. The frequency of drinking option ≥4 days weekly converts to 22 days for each four weeks, and the typical quantity option ≥10 drinks converts to 100 g of alcohol. Therefore, the actual difference in baseline total alcohol consumption between groups might be larger than indicated by AUDIT-C scores. However, we included baseline total alcohol consumption, converted from AUDIT-C scores, as a covariate in the efficacy analysis, accounting for this difference. Furthermore, subgroup analysis did not suggest the superiority of the ultra-brief intervention group in any category, including the highest AUDIT-C score category, where the ceiling effect would have had the most impact on the results.

### Generalisability

The generalisability of our findings can be considered from two perspectives, with our results likely extending beyond Japan to other populations. Japan is characterised by a culture of permissive alcohol consumption, where public drinking is highly socially accepted.^46^ However, the proportion of people who consume alcohol at levels exceeding the non-drinker equivalence level—where the risk of harm to health equals that of non-drinkers— is similar between Japan and western countries, including the UK, western Europe, and north America. For example, among adults aged 40-64 years, this proportion was 43.9% for men and 55.6% for women in Japan, compared with 30.7-52.9% for men and 44.3-61.5% for women in these western countries.^47^ Therefore, our findings might be generalisable to western contexts.

Our findings would similarly apply to healthcare settings with more established intervention practices for hazardous drinking. In Japanese primary care, alcohol screening and brief interventions are not routinely implemented, as evidenced by the present trial’s lack of excluded clinics based on existing practices, providing a neutral setting to assess the effectiveness of ultra-brief intervention without background interventions. In healthcare systems where more intensive interventions are standard practice,^48^ ^49^ ^50^ the potential impact of ultra-brief intervention would be further diminished, as any modest effects would be masked by existing interventions. Thus, the lack of supporting evidence for the effectiveness of ultra-brief intervention in a setting without routine interventions would likely apply to contexts where more intensive baseline interventions are already established.

### Strengths and limitations of this trial

This trial has several strengths. The screening rates at the cluster level were high (median >70% *v* 5% in SIPS trial^10^), maintaining the pragmatic nature of our study and strengthening external validity. The high follow-up rates (84%) minimised the risk of attrition bias that could threaten internal validity. The control condition closely resembled real world settings of no screening and intervention through simplified study procedures and questionnaires, a critical feature when evaluating small intervention effects like those hypothesised for ultra-brief intervention. Our large sample size (n=1133) provided sufficient statistical power to detect clinically meaningful differences between groups, which is particularly important in a trial such as ours with null findings.

Furthermore, previous systematic reviews of brief interventions in these settings included only a few trials from an Asian country,^8^ ^51^ with Asians comprising merely 1% of participants in western studies.^8^ While the proportion of people drinking above the health risk equivalence threshold of non-drinkers in Japan was higher than that of other Asian countries,^47^ our findings remain important for Asian populations where such data have been notably scarce. These strengths contribute to a nuanced understanding of the intervention’s potential clinical impact in real world settings.

However, some limitations should be acknowledged. Baseline data on total alcohol consumption and readiness to change drinking behaviour were not collected. Although this was to avoid the potential effect of intensive screening, it became difficult to balance between group differences in these outcomes at baseline due to random error.

We used simple questions to measure total alcohol consumption in the four weeks preceding the 12 week and 24 week follow-ups instead of more comprehensive measures like drinking diaries or timeline followback. While this approach might introduce recall bias, such bias would likely affect both groups equally, preserving the validity of between group comparisons. This approach aligns with other trials of brief intervention that similarly used self-reported frequency of drinking and amounts consumed on each typical drinking occasion rather than intensive assessments.^52^ ^53^ ^54^ We believe this was a realistic approach for detecting the effect of ultra-brief intervention rather than using these intensive assessment methods as potential co-interventions.

Reliance on self-reported invitation and participation rates from the clinic clusters, as well as their self-reported adherence to the intervention protocol without direct observation, may have introduced bias towards no effect. However, strict adherence monitoring could differ from daily practice.

Finally, 64% of participants in the ultra-brief intervention group reported receiving advice from their doctors, which might indicate incomplete delivery of the intervention. However, this proportion could reflect the subtle nature of ultra-brief intervention rather than non-adherence of the doctors to delivery, as similar recognition rates were observed even in our pilot trial where ultra-brief intervention was delivered to 100% of participants. While this factor could potentially bias results towards the null, our trial evaluated a policy estimand that measured the effectiveness of telling doctors to implement ultra-brief intervention rather than the efficacy of perfectly delivered intervention. Furthermore, the sensitivity analysis accounting for non-adherence of doctors also showed no evidence of benefit for the ultra-brief intervention group.

### Implications

Despite these limitations, this trial has important implications for alcohol screening and brief interventions in primary care. From a public health perspective, our results suggest that widely implementing ultra-brief intervention in primary care may not lead to clinically relevant benefits to reduce alcohol consumption. However, from a clinical standpoint, some doctors may recognise the importance of the ultra-brief intervention based on its impact on readiness to change drinking behaviour, given that the strength of primary care lies in continuity of care. Informed by feedback from PPI, future research should focus on developing both feasible and effective interventions for primary care settings—where many patients with alcohol problems first seek care. These efforts should also be directed towards investigating the mechanisms of action through which interventions influence drinking behaviour and assessing outcomes beyond harm to patients themselves, including the effect on family members and society.

### Conclusion

This study found no evidence to support the superiority of ultra-brief intervention over simplified assessment only in reducing alcohol consumption among patients with hazardous drinking or suspected alcohol dependence in Japanese primary care settings.

**What is already known on this topic**

Brief interventions for hazardous drinking have been widely recommended in primary care settings, but implementation rates remain low owing to various barriers
Ultra-brief interventions have shown mixed results in different settings, with some studies suggesting they can be as effective as longer advice or counselling
No randomised controlled trial has directly investigated the effectiveness of ultra-brief interventions over assessment only control in primary care settings

**What this study adds**

This large scale pragmatic cluster randomised controlled trial found no evidence to support the effectiveness of ultra-brief intervention in reducing alcohol consumption compared with simplified assessment only in primary care settings in Japan
Participants in the ultra-brief intervention group showed higher readiness to change drinking behaviour at 12 weeks and 24 weeks compared with simplified assessment only, despite not reducing alcohol consumption
These findings challenge the syllogistic reasoning that ultra-brief intervention would be effective because standard brief intervention is effective and because ultra-brief intervention is as effective as standard brief intervention

## Data Availability

The datasets and statistical codes can be found at https://doi.org/10.5061/dryad.866t1g22m.

## Web extra

Extra material supplied by authors

Supplementary file 1: Ultra-brief intervention leaflet (Japanese)

Supplementary file 2: Ultra-brief intervention leaflet (English)

Supplementary file 3: Ultra-brief intervention provider instructions (Japanese)

Supplementary file 4: Ultra-brief intervention provider instructions (English)

## Notes

Contributors: RS was responsible for study conceptualisation, methodology, formal analysis, data curation, project administration, and funding acquisition, and drafted the original manuscript. KK, SM, KN, YH, HY, and TY contributed to the study conceptualisation and methodology. SM and KN were also involved in funding acquisition. SO contributed to methodology, formal analysis, resources, and data curation. YT, TS, and HK provided methodological support and resources. MK, SN, and YT conducted the investigation. HN was responsible for visualisation. TAF provided supervision. ES, HK, and YO contributed to the critical review and editing of the manuscript. All authors contributed to the writing, review, and editing of the manuscript. All authors had full access to all of the study data and take responsibility for the integrity of the data and the accuracy of the data. RS is the guarantor. The corresponding author attests that all listed authors meet authorship criteria and that no others meeting the criteria have been omitted. During the preparation of this manuscript, we used chatGPT, Claude, and Grammarly for proofreading and sentence revision of the English language but not for their intellectual content. After using these tools, we thoroughly reviewed and edited the content where necessary. We assume full responsibility for the content of the publication.

Transparency: The lead author (RS), the manuscript’s guarantor, affirms that the manuscript is an honest, accurate, and transparent account of the study being reported; that no important aspects of the study have been omitted; and that any discrepancies from the study as planned (and, if relevant, registered) have been explained.

Dissemination to participants and related patient and public communities: We will disseminate our findings through several channels. Since the ultra-brief intervention is not yet widely implemented in Japan, and our study found null results, we will focus primarily on informing researchers and policy makers rather than the general public. We plan to present our findings at scientific conferences to reach healthcare researchers. Additionally, we will seek to present at the conference of a cross party parliamentary group on alcohol related problems to inform policy decisions through network of ASK, that provided feedback on our manuscript as an aspect of patient and public involvement.

Provenance and peer review: Not commissioned; externally peer reviewed.
