# Supplementary material for: Effectiveness of screening and ultra-brief intervention for hazardous drinking in primary care: pragmatic cluster randomised controlled trial
Source: BMJ. 2025 Aug 12;390:e083985. doi: 10.1136/bmj-2024-083985 (PMC12340667; doi:10.1136/bmj-2024-083985)
Supplement: Supplementary file 1 — Ultra-brief intervention leaflet (Japanese) [file sory083985.ww1.pdf]

多忙な「医療スタッフ、産業保健スタッフ」向け

ver. 1.1
令和２年３月作成

**30秒でできる！**

# 「超」簡易減酒支援
# Ultra-brief Intervention

**対象** 飲酒量に問題がありそうな人

**方法** リーフレットを渡して、内容を短く伝えるだけ

| | |
|---|---|
| フィードバック | ○○さん、どうやら飲酒量が多いようです。 |
| アドバイス | ぜひご自身で飲酒量を計算してみてください。 |
| 効果を保証 | このリーフレットは○○さんに必ず役に立つはずです！ 読むだけで飲酒量が減った人がいます。 |
| できることを保証 | 今日からでもすぐにできる内容です！ |
| 約束により動機づけ | 次にお会いした時にぜひ感想を聞かせてください♡ |

**POINT 1**
**効果が期待できる人**
❶信頼関係ができている患者さん（外来でも入院でも）
❷同じ職場で何度も会える人

**POINT 2**
**努力している場合**
次回、様子を聞いて努力している場合などには、「頑張っていますね」と一緒に喜びましょう。

**POINT3**
**反論された場合**
「飲み過ぎって言われたけれど、そうでもなかったよ」などと反論されたら、説得せずに声がけを。
❶「よかったですね！」
❷「今後のためにも用心してくださいね」

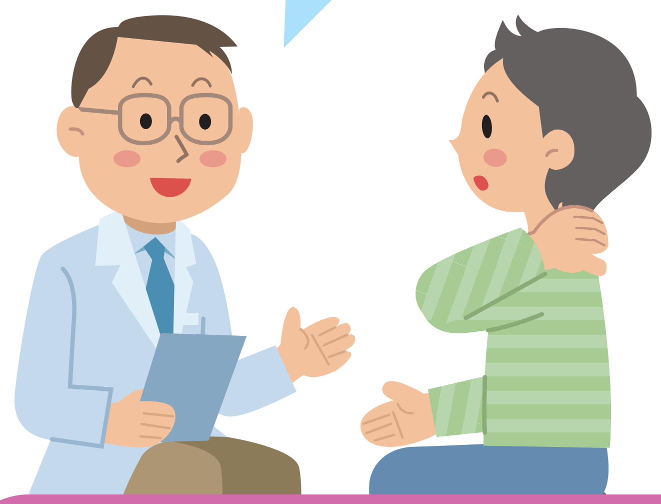

は、はぁ…

**とまどいつつも診察後や会計前などの待ち時間に**

**リーフレットに目を通す**

↓

**実際に行動が変わる**

**これだけで終了です！**

この支援法で飲酒量が平均２ドリンク（ビール500ml相当）減少することが臨床研究（RCT）により示されました。

# 1 より効果的な声かけは？

## 1. 具体的に飲酒量をフィードバック

○○さんの飲酒量は 8 ドリンクです（一緒に計算）。

8 ドリンクは 6 ドリンクを超えているので多量飲酒です。赤信号ですね。

## 2. 具体的な目標をたてる

（リーフレットの 3 ページを一緒に見ながら）
まずは無理せずできそうな目標を立ててみましょう。

## 3. 具体的な対処法

（リーフレットの 3・4 ページを一緒に見ながら）
何か生活に取り入れられそうな工夫はありますか。

- できるだけ具体的に話すことがポイントです。

- 支援者が目標や対処法を決めるのではなく、自分自身で決めてもらうことにより、さらに効果的になります。
- 実際にリーフレットに記入してもらいましょう！

- 目標を立てたら、記録を勧めましょう！

# 2 より具体的な飲酒問題の評価法

## 対象▶飲酒量に問題がありそうな人

飲酒問題があるかどうかの指標として AUDIT-C があります。AUDIT-C は、飲酒頻度・1 回飲酒量・多量飲酒頻度の 3 項目を聞くだけなので、簡便で実践的です。

# 3 5分以上の時間が確保できる場合

より効果的な減酒支援の方法として、ABCD プログラムや HAPPY プログラムといった双方向のやりとりによる「簡易介入 Brief Intervention」があります。ぜひ実践してみましょう。

# 4 介入しても減酒できない or 増える場合

アルコール依存症の可能性があります。AUDIT（10 項目）によるスクリーニングやアルコール依存症の診断基準（ICD-10）を参考にしてください。依存症を疑う場合は、専門医療機関への紹介をご検討ください。専門医療機関以外でもできることとして、ABCDE プログラムがあります。

資料のダウンロードは、検索または QR コードから！

依存症対策全国センター　簡易介入　［検索］

AMED「アルコール依存症予防のための簡易介入プログラム開発と効果評価に関する研究」（研究代表者：杠岳文）
本資料に関する問い合わせ先：国立病院機構琉球病院　手塚幸雄／ubi.alcohol@gmail.com
＊本テキストは著作権を放棄しており、複製、公衆送信、翻訳等の翻案等、自由にご利用いただけます。使用にあたっての連絡は必要ありません。
