# Supplementary material for: Effectiveness of screening and ultra-brief intervention for hazardous drinking in primary care: pragmatic cluster randomised controlled trial
Source: BMJ. 2025 Aug 12;390:e083985. doi: 10.1136/bmj-2024-083985 (PMC12340667; doi:10.1136/bmj-2024-083985)
Supplement: Supplementary file 2 — Ultra-brief intervention leaflet (English) [file sory083985.ww2.pdf]

For medical & occupational health staff

ver. 1.1
Developed in March 2020.

30-second ! Ultra-Brief Intervention

Target For those with potential drinking issues

Methods Give the patient a brochure & briefly explain.

Feedback	Mr./Ms. [Name], you may be drinking too much.
Advice	I recommend you calculate your alcohol consumption on your own.
Guarantee of effectiveness	This brochure will help you! Some people reduced their drinking just by reading this.
Assurance of feasibility	You can start these changes today!
Commitment-based motivation	Please share your thoughts next time we meet!

POINT 1

Who can benefit.

❶ Patients with established trusting relationship (outpatient/inpatient)

❷ In occupational health settings, participants receive feedback from people they see daily at work.

POINT 2

If patient is making an effort.

Next time, celebrate: “You’re doing great!”

POINT3

When faced with objections.

If they say, “I’m not drinking much,” don’t argue.

Say, ”Great! Stay mindful!”

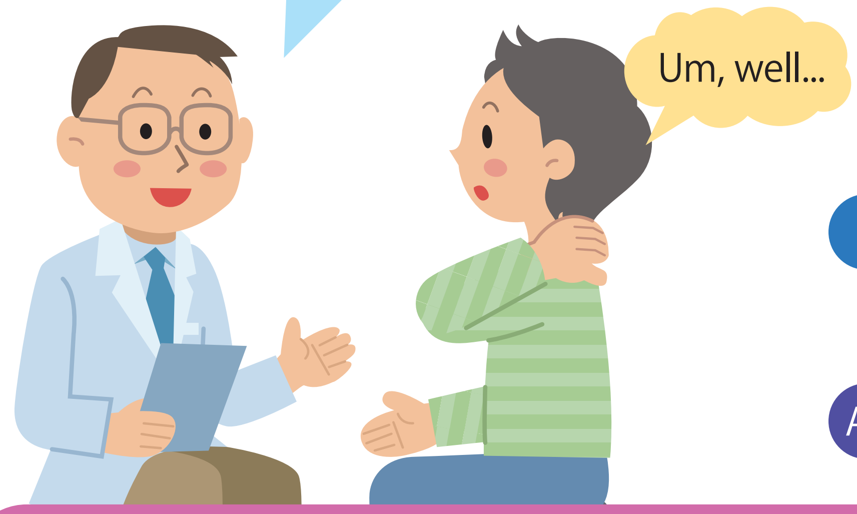

If patients still resist, ask them to read the brochure in the waiting area.

Patient reads brochure

↓

Actual behavior change occurs

That’s all you need to do! ▶ That’s it! Research shows this can cut drinking by 2 drinks per week (500ml beer)

1 What are more effective approaches?

1. Give specific feedback on alcohol consumption.

[Name], you drank 8 drinks (calculated together).

Over 6 drinks is considered heavy drinking.
That's a red flag.

2. Set specific goals.

(Looking at page 3 of the brochure together)
First, let's set achievable goals together.

3. Specific coping strategies.

(Check pages 3-4 of the brochure)
What change could you make in your daily life?

- The key is to be as specific as possible.

- Let them choose their own goals and methods - it's more effective than deciding for them.
- Have them write in the brochure!

- After setting goals, encourage recordkeeping.

2 A more concrete evaluation of drinking problems

Target ▶ Those with potential drinking issues

AUDIT-C helps identify drinking problems.
AUDIT-C: Just 3 Qs - frequency, amount, heavy drinking, it's quick & practical

3 If you have more than 5 minutes

For more effective alcohol reduction support, try ABCD and HAPPY interactive Brief Intervention programs.

4 If drinking persists/worsens after intervention

Consider alcohol dependence - Use AUDIT (10 items) or diagnostic evaluation for alcohol dependence (ICD-10). If alcohol dependence is suspected, refer to specialists. Try the ABCDE program if specialist care is unavailable.

Download materials via search or QR code!

National Center for Addiction Services Administration | Search

AMED "Development and Evaluation of a Brief Intervention Program for Alcohol Dependence Prevention" (Principal Investigator: Takefumi Yuzuriha)
Contact: Yukio Tezuka, NHO Ryukyu Hospital / ubi.alcohol@gmail.com
*Free to copy, distribute, translate. No permission needed.
