# Supplementary material for: Effectiveness of screening and ultra-brief intervention for hazardous drinking in primary care: pragmatic cluster randomised controlled trial
Source: BMJ. 2025 Aug 12;390:e083985. doi: 10.1136/bmj-2024-083985 (PMC12340667; doi:10.1136/bmj-2024-083985)

7 酒席ではコップを空にしない（注がれないため）

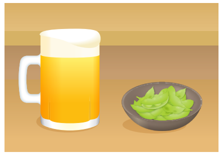

8 上手な断り方を身につける

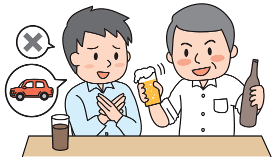

9 お酒を飲みすぎてしまう相手や場所を避ける

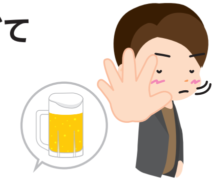

10 お酒を飲むと検査値が悪くなることを思い浮かべる

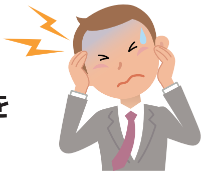

11 お酒を減らすと健康になることを思い浮かべる

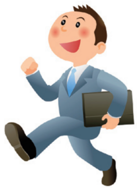

12 お酒以外の楽しみや趣味を増やす

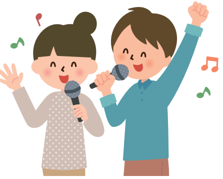

13 周りの人に目標を宣言して協力してもらう

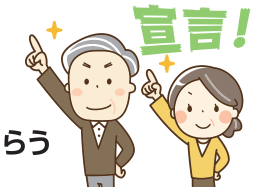

14 家族や友人と楽しく過ごす

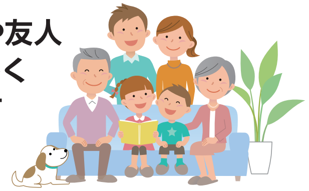

ドリンク数の記録のために、以下のサイト・アプリもオススメです！

飲酒チェックツール SNAPPY-CAT

あなたのお酒の飲み方について、約３分間で振り返りができます。

また、あなたの飲酒量を簡単に調べることができます。

検索サイトで

SNAPPY-CAT 検索 と検索！

https://www.udb.jp/snappy_test/

どの都道府県の方にもおススメ！

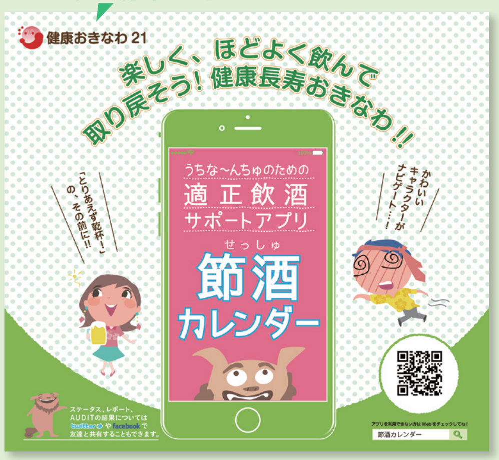

お酒と上手に付き合って、楽しく健康的な生活をおくりましょう！

AMED「アルコール依存症予防のための簡易介入プログラム開発と効果評価に関する研究」（研究代表者：杠岳文）
本資料に関する問い合わせ先：国立病院機構琉球病院　手塚幸雄／ubi.alcohol@gmail.com
＊本テキストは著作権を放棄しており、複製、公衆送信、翻訳等の翻案等、自由にご利用いただけます。使用にあたっての連絡は必要ありません。

お酒との上手な付き合い方

ver. 1.1
令和２年３月作成

このリーフレットは、減酒が望ましいと思われる方向けのものです。きっと役立つ内容ですので、ぜひお読みください。食事には、「カロリー」という、共通の物差しがあるように、お酒にも、「ドリンク」という、共通の物差しがあります。ドリンクを覚えると、自分がどれだけのアルコールを飲んでいるかが分かりやすくなります。

ドリンク換算表

ビール (5%)	日本酒 (15%)	缶チューハイ (7%)	泡盛・焼酎 (30%)	ワイン (12%)	ウイスキー (40%)
500ml 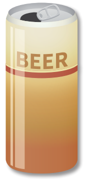	1合 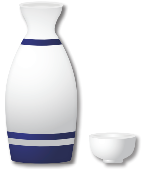	350ml 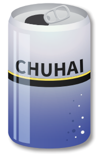	1合 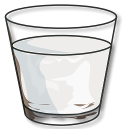	グラス1杯 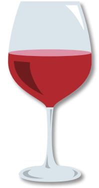	ダブル1杯 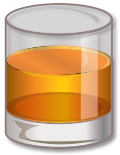
2 ドリンク	2 ドリンク	2 ドリンク	4 ドリンク	1 ドリンク	2 ドリンク

例えば 缶ビール（500ml）を1本飲んで、そのあとに日本酒を1合飲むとすると……

缶ビール 2ドリンク 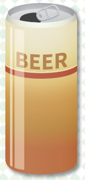 ＋ 日本酒 2ドリンク 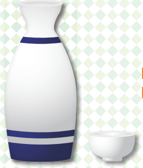 ＝ 4 ドリンク

となります。あなたのいつものお酒は何ドリンクでしょうか？
ぜひ計算してみてください！

あなたのいつものお酒は ＿＿＿＿ ドリンク

飲酒量「ドリンク」の目安（1日あたり）

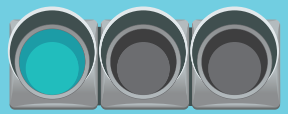

2ドリンク以下

適度な飲酒量

今の習慣を続けましょう。女性は**1**ドリンクが目安です。休肝日を**週に3日**以上つくりましょう。

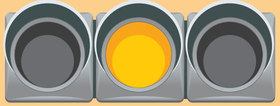

男性4ドリンク
女性2ドリンク

生活習慣病のリスクを高める量

糖尿病、高血圧、脂質異常症、痛風、肥満になるリスクが高まります。すでに病気がある人は、飲酒による悪影響が……！？

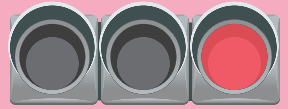

6ドリンク以上

多量飲酒

数年で肝硬変やアルコール依存症などにかかる危険があります。

（黄）&（赤）の方は **お酒を減らすことをオススメします！**

このまま
お酒の飲みすぎが続くと……

お酒を楽しめない生活になってしまいます

こんな病気の危険が！

- 脳出血
- 脳梗塞
- 心筋梗塞
- 肝硬変
- 痛風
- アルコール依存症

など

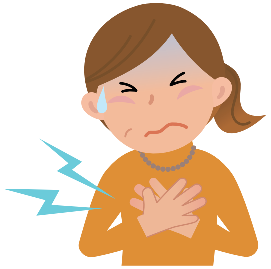

ここで
お酒を減らすことで……

健康的な生活を続けてお酒も楽しめます

こんな健康効果が！

- 血圧が下がる
- 体重が減る
- 肝機能が改善する
- 血糖値が下がる
- 尿酸値が下がる

など、ほかにも色々よいことがあります

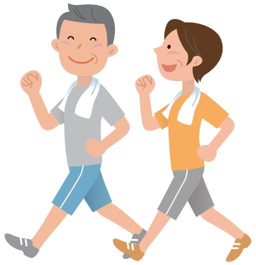

お酒を楽に減らすには

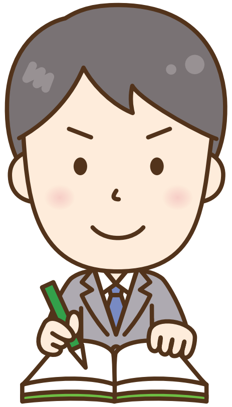

① 自分のドリンク数を、毎日記録する

日記、手帳、カレンダーなど毎日、目にするものに記録するのがオススメです。つぎのページの2つのアプリは手軽で便利です♪

② 減酒目標を立てる

まずは「**1日1ドリンク減らす**」か「**休肝日を1日増やす**」など、できるところから始めてみましょう。無理はしないで。

③ 目標達成できたかチェックする

うまくできた：○　まあまあ：△　できなかった：×　などで自己評価します。

わたしの目標は　　　　　　　　　　　**です。**

さあ、今日から目標達成に向かって始めましょう！

お酒を飲みすぎない対処法

1 まず、お腹を満たす

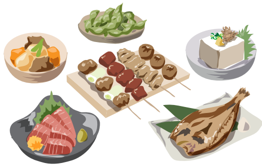

2 お酒を飲む曜日を決めておく

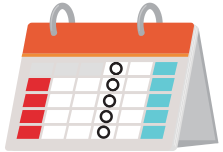

3 ノンアルコール飲料を上手に活用する

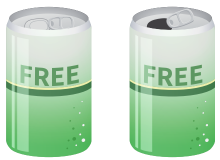

4 寝酒は止める（眠りが浅くなる）

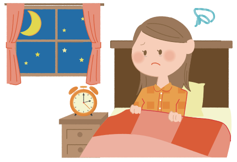

5 1口飲むたびにコップをテーブルに置く

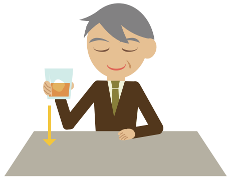

6 1日3時間以上飲まない

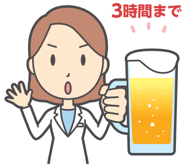

Supplement: Supplementary file 3 — Ultra-brief intervention provider instructions (Japanese) [file sory083985.ww3.pdf]
