# Supplementary material for: Effectiveness of screening and ultra-brief intervention for hazardous drinking in primary care: pragmatic cluster randomised controlled trial
Source: BMJ. 2025 Aug 12;390:e083985. doi: 10.1136/bmj-2024-083985 (PMC12340667; doi:10.1136/bmj-2024-083985)

7 Keep your glass partly full at parties (to avoid refills).

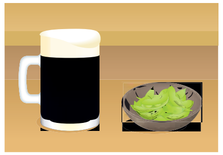

8 Learn to politely say "no."

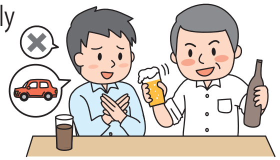

9 Avoid heavy-drinking friends/places.

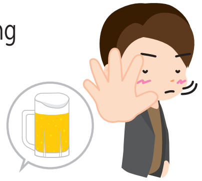

10 Remember how alcohol worsens your test results.

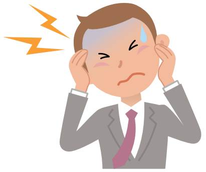

11 Think about how reducing alcohol improves your health.

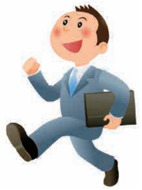

12 Find non-drinking hobbies/activities.

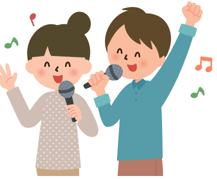

13 Share your goals with those around you for support.

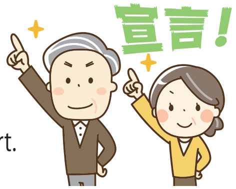

14 Enjoy time with family/friends.

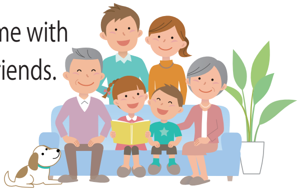

For tracking your drinks, these sites/apps are recommended!

Drinking Check Tool: SNAPPY-CAT

Review your drinking habits in about 3 minutes.

Easily check your alcohol consumption.

Search for "SNAPPY-CAT" online!

SNAPPY-CAT Search

https://www.udb.jp/snappy_test/

Recommended for everyone nationwide!

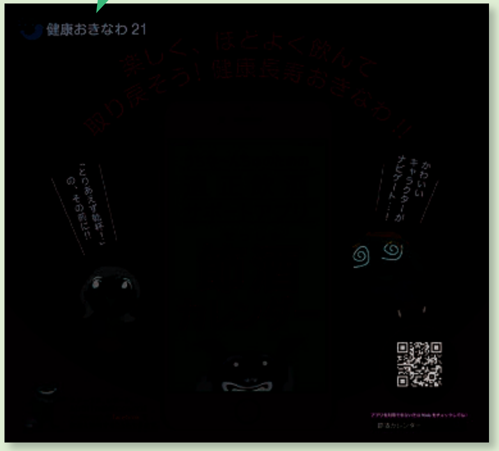

Enjoy a healthy lifestyle by drinking responsibly!

AMED "Development and Evaluation of a Brief Intervention Program for Alcohol Dependence Prevention" (Principal Investigator: Takefumi Yuzuriha)

Contact: Yukio Tezuka, NHO Ryukyu Hospital / ubi.alcohol@gmail.com

*Free to copy, distribute, translate. No permission needed.

Smart Drinking Guide

ver. 1.1
Developed March 2020

This brochure is intended for those who may benefit from reduced drinking. You'll find it helpful - please read on. Just as food has "calories", alcohol has "standard drinks" as a measure. Thinking about "standard drinks" makes it easier to understand the amount of alcohol intake.

Drink Conversion Chart

(gō is a standard measure equal to 180 ml of sake in Japan)

Beer (5%)	Sake (15%)	Chūhai (7%)	Shōchū (30%)	Wine (12%)	Whiskey (40%)
500ml	1 gō	350ml	1 gō	1 glass	1 double
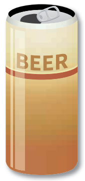	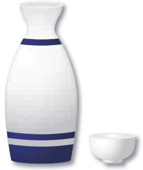	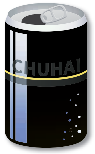	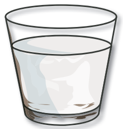	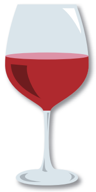	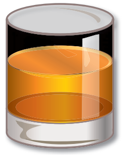
2 drinks	2 drinks	2 drinks	4 drinks	1 drink	2 drinks

For example, If you drink one can of beer (500ml) and then one gō of sake...

Beer 2 drinks 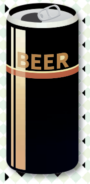 + Sake 2 drinks 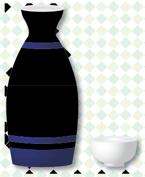 = 4 drinks

How many drinks of alcohol do you normally have? Try calculating it!

Your regular alcohol intake is ______ drinks.

Recommended"Drink"Amounts (Per Day)

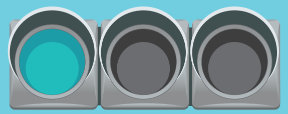

2 drinks or less

Moderate drinking

Continue your current habits. For women, 1 drink is recommended. Establish at least 3 alcohol-free days per week.

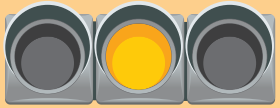

Men: 4 drinks
Women: 2 drinks

Raises lifestyle disease risk

Increases risk of diabetes, hypertension, dyslipidemia, gout, and obesity. If you already have these conditions, alcohol can worsen them!

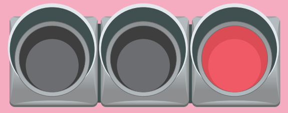

6 drinks or more

Hazardous drinking

Risk of cirrhosis and alcohol dependence within years.

If you're in the (yellow) & (red) zone: we recommend reduced drinking.

If excessive drinking continues...

You'll be unable to enjoy alcohol at all.

Health risks include:

- Cerebral hemorrhage
- Cerebral infarction
- Myocardial infarction
- Cirrhosis
- Gout
- Alcohol dependence

And more

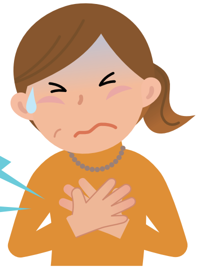

By reducing alcohol now...

You can stay healthy while still enjoying alcohol.

Health benefits include:

- Lower blood pressure
- Weight loss
- Lower blood sugar
- Improved liver function
- Lower uric acid

And many more positive effects

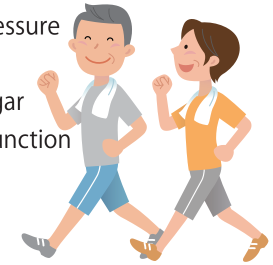

Easy Ways to Reduce Your Drinking

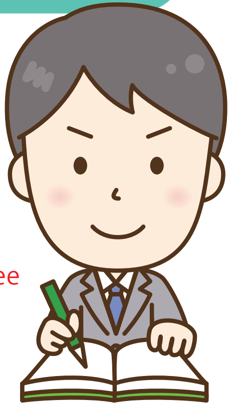

1 Track your drinks daily.

Record in a diary, planner, or calendar - something you see daily. Two apps on the next page are easy to use!

2 Set a drinking reduction goal.

Start small: "One less drink per day" or "One more alcohol-free day." Don't push yourself too hard.

3 Check if you've achieved your goal.

Rate yourself: ○ = Success, △ = Moderate success, × = Unsuccessful

My goal is ______________________.

Let's start working toward your goal today!

Tips to Avoid Drinking Too Much

1 Eat before drinking.

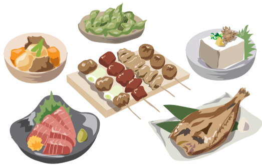

2 Decide which days you'll drink.

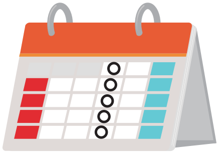

3 Use non-alcoholic beverages wisely.

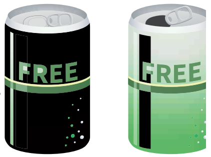

4 Stop nightcaps (they block deep sleep).

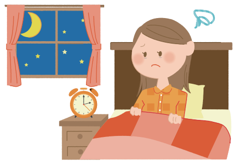

5 Put your glass down between sips.

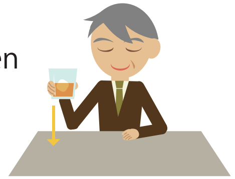

6 Don't drink for more than 3 hours in a day.

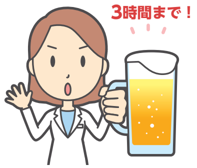

Supplement: Supplementary file 4 — Ultra-brief intervention provider instructions (English) [file sory083985.ww4.pdf]
